# Synthesis and preclinical evaluation of [^11^C]EAI045 as a PET tracer for imaging tumors expressing mutated epidermal growth factor receptor

**DOI:** 10.1186/s13550-024-01078-6

**Published:** 2024-02-16

**Authors:** Antonia A. Högnäsbacka, Alex J. Poot, Christophe Plisson, Jonas Bergare, David R. Bonsall, Stuart P. McCluskey, Lisa A. Wells, Esther Kooijman, Robert C. Schuit, Mariska Verlaan, Wissam Beaino, Guus A. M. S. van Dongen, Danielle J. Vugts, Charles S. Elmore, Jan Passchier, Albert D. Windhorst

**Affiliations:** 1 Department Radiology and Nuclear Medicine, Vrije Universiteit Amsterdam, Amsterdam UMC, De Boelelaan 1117, 1081HV Amsterdam, The Netherlands; 2 Cancer Center Amsterdam, Biomarkers and Imaging, Amsterdam, the Netherlands; 3 Invicro LLC, London, W12 0NN UK; 4 Early Chemical Development, Pharmaceutical Sciences, R&D AstraZeneca, Gothenburg, Sweden

**Keywords:** EAI045, Epidermal growth factor receptor, EGFR, Tyrosine kinase inhibitor, TKI

## Abstract

**Background:**

Mutations in the epidermal growth factor receptor (EGFR) kinase domain are common in non-small cell lung cancer. Conventional tyrosine kinase inhibitors target the mutation site in the ATP binding pocket, thereby inhibiting the receptor's function. However, subsequent treatment resistance mutations in the ATP binding site are common. The EGFR allosteric inhibitor, EAI045, is proposed to have an alternative mechanism of action, disrupting receptor signaling independent of the ATP-binding site. The antibody cetuximab is hypothesized to increase the number of accessible allosteric pockets for EAI045, thus increasing the potency of the inhibitor. This work aimed to gain further knowledge on pharmacokinetics, the EGFR mutation-targeting potential, and the influence of cetuximab on the uptake by radiolabeling EAI045 with carbon-11 and tritium.

**Results:**

2-(5-fluoro-2-hydroxyphenyl)-2-((2-iodobenzyl)amino)-*N*-(thiazol-2-yl)acetamide and 2-(5-fluoro-2-hydroxyphenyl)-*N*-(5-iodothiazol-2-yl)-2-(1-oxoisoindolin-2-yl)acetamide were synthesized as precursors for the carbon-11 and tritium labeling of EAI045, respectively. [^11^C]EAI045 was synthesized using [^11^C]CO in a palladium-catalyzed ring closure in a 10 ± 1% radiochemical yield (decay corrected to end of [^11^C]CO_2_ production), > 97% radiochemical purity and 26 ± 1 GBq/µmol molar activity (determined at end of synthesis) in 51 min. [^3^H]EAI045 was synthesized by a tritium-halogen exchange in a 0.2% radiochemical yield, 98% radiochemical purity, and 763 kBq/nmol molar activity. The ability of [^11^C]EAI045 to differentiate between L858R/T790M mutated EGFR expressing H1975 xenografts and wild-type EGFR expressing A549 xenografts was evaluated in female nu/nu mice. The uptake was statistically significantly higher in H1975 xenografts compared to A549 xenografts (0.45 ± 0.07%ID/g vs. 0.31 ± 0.10%ID/g, *P* = 0.0166). The synergy in inhibition between EAI045 and cetuximab was evaluated in vivo and in vitro. While there was some indication that cetuximab influenced the uptake of [^3^H]EAI045 in vitro, this could not be confirmed in vivo when tumor-bearing mice were administered cetuximab (0.5 mg), 24 h prior to injection of [^11^C]EAI045.

**Conclusions:**

EAI045 was successfully labeled with tritium and carbon-11, and the in vivo results indicated [^11^C]EAI045 may be able to distinguish between mutated and non-mutated EGFR in non-small cell lung cancer mouse models. Cetuximab was hypothesized to increase EAI045 uptake; however, no significant effect was observed on the uptake of [^11^C]EAI045 in vivo or [^3^H]EAI045 in vitro in H1975 xenografts and cells.

**Supplementary Information:**

The online version contains supplementary material available at 10.1186/s13550-024-01078-6.

## Background

Treatment success of Epidermal Growth Factor Receptor (EGFR) mutated Non-Small Cell Lung Cancer (NSCLC) with Tyrosine Kinase Inhibitors (TKIs) is hampered by treatment resistance development [1, 2]. Multiple TKIs have been developed for the treatment of the most common primary mutations, L858R and Del19 [3]. Treatment resistance against these inhibitors inevitably ensues, which restores the constitutive EGFR-dependent signaling, most often in the form of the tyrosine kinase T790M mutation [4, 5]. TKIs able to inhibit the T790M mutation have been developed, but additional treatment resistance in the form of the C797S mutation is common [6]. Jia et al. hypothesized that further development of resistance mutations at the ATP-binding site could be circumvented by inhibition at an allosteric pocket in the receptor. EAI045, an allosteric inhibitor selective for the L858R/T790M EGFR mutation, was therefore developed [7].

When the potency of EAI045 to inhibit L858R/T790M mutated EGFR in H1975 cells was evaluated, it was found that EAI045 could not completely inhibit EGFR auto-phosphorylation. Jia et al*.* postulated that the allosteric pocket of one of the receptors in the dimeric EGFR complex is inaccessible to EAI045. This theory was examined by using EAI045 in combination with cetuximab, an antibody that disrupts the ligand binding and the dimer formation of EGFR. A synergistic effect of the increased potency of EAI045 was observed both in vitro in murine cells and in vivo in a genetically engineered mouse model bearing murine lung carcinomas induced by L858R/T790M mutant EGFR [7].

Many EGFR TKIs have been radiolabeled with positron emission tomography (PET) isotopes and used as a tool to evaluate the inhibitor's pharmacokinetics and mutational selectivity, as radiolabeled compounds offer high sensitivity and accuracy in detection [8]. In this work, racemic EAI045 is labeled using carbon-11 and tritium to gain more insights into the pharmacokinetic characteristics of EAI045, its selectivity for mutated EGFR, and the influence of cetuximab on the uptake of EAI045 in EGFR-expressing cells. The capability of [^11^C]EAI045 to distinguish in vivo between wild-type and L858R/T790M mutated EGFR was evaluated in female nu/nu mice bearing human NSCLC xenografts. In addition, the synergistic influence of cetuximab was evaluated in vivo using [^11^C]EAI045 and in vitro using [^3^H]EAI045.

## Results

### Chemistry

#### [^3^H]EAI045

**Scheme 1:**
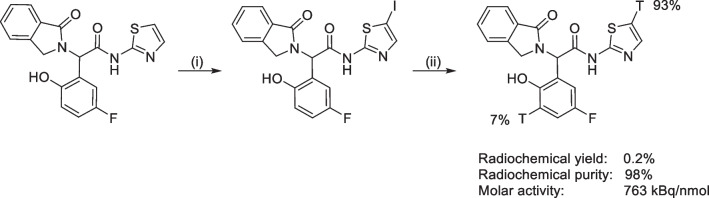
Iodination of EAI045, followed by the tritiodehalogenation: (i) *N*-iodosuccinimide, trifluoroacetic acid, dichloromethane, room temperature, 2.5 h; (ii) tritium gas, palladium on calcium carbonate, ethanol, room temperature, 4 h

EAI045 was labeled with tritium via a tritiodehalogenation. The precursor was obtained via iodination of EAI045 with N-iodosuccinimide in dichloromethane. Subsequently, the tritiodehalogenation was achieved by reacting 2-(5-fluoro-2-hydroxyphenyl)-*N*-(5-iodothiazol-2-yl)-2-(1-oxoisoindolin-2-yl)acetamide with tritium gas in ethanol containing palladium on calcium carbonate (Scheme 1) which yielded [^3^H]EAI045 in a radiochemical yield of 0.2%, a radiochemical purity of 98%, and a molar activity of 763 kBq/nmol. ^3^H NMR analysis showed 93% of the label to be located as expected in the thiazole ring; however, 7% of the tritium was located on the aromatic ring next to the phenol. One of the byproducts in the precursor preparation was a di-iodinated compound. Therefore, it is possible that one of the batches of freeze-dried precursor had traces of di-iodinated precursor, which subsequently resulted in [^3^H]EAI045 labeled with tritium in two positions, 2-(5-fluoro-2-hydroxyphenyl-3-^3^H)-2-(1-oxoisoindolin-2-yl)-*N*-(thiazol-2-yl-5-^3^H)acetamide (Scheme 1).

#### [^11^C]EAI045

A carbon-11 containing isotopologue of EAI045 was synthesized to enable in vivo evaluation. The choice of labeling position was based on previous research by Karimi et al. [9]. However, in contrast to their work, the amine in the current study was secondary, making it considerably less reactive.

### Synthesis of precursor

**Scheme 2:**
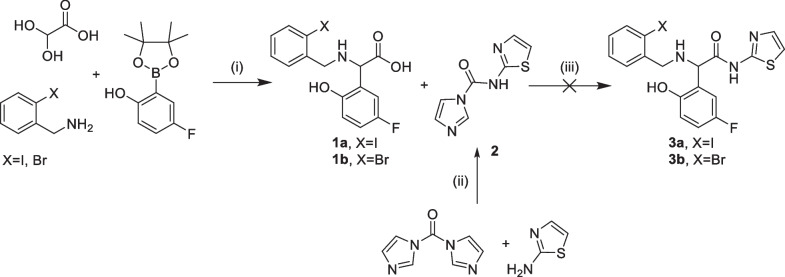
Petasis approach for the synthesis of [^11^C]EAI045 precursor, (i) methanol, room temperature, 22 h, 29%; (ii) dichloromethane, room temperature, 2.5 h, 56%; (iii) dichloromethane, room temperature, 19 h, 0%

The synthesis of precursor **3** for radiolabeling with ^11^C was first attempted by a Petasis reaction [10]. However, the subsequential amide condensation step proved to be challenging. Neither the use of condensation reagents (not shown) nor the use of a carbonyldiimidazole [11] activated 2-aminothiazole yielded the desired compound (Scheme 2).

**Scheme 3:**
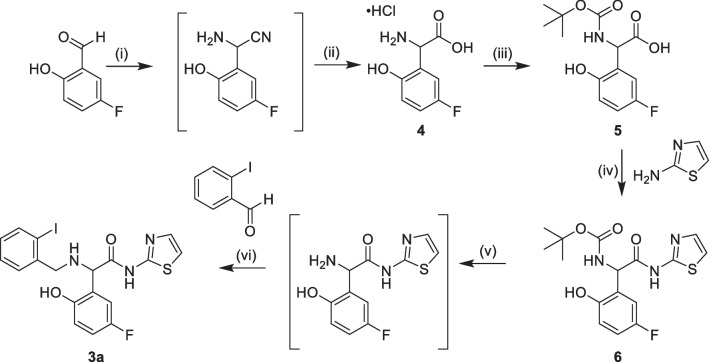
Synthesis of [^11^C]EAI045 precursor (**3a**): (i) trimethylsilyl cyanide, ammonia in methanol, 45 °C, 30 min; (ii) hydrochloric acid in water, reflux/room temperature, overnight; (iii) di-*tert*-butyl dicarbonate, sodium hydroxide, water/*tert*-butanol, room temperature, overnight, 5% yield over three steps; (iv) *N,N*-diisopropylethylamine, propylphosphonic anhydride, ethyl acetate/dimethylformamide, room temperature, overnight, 15% yield; (v) trifluoroacetic acid, dichloromethane, room temperature, 15 min; (vi) 1,8-diazabicyclo(5.4.0)undec-7-ene, sodium sulfate, methanol, 55 °C, 1 h, Sodium cyanoborohydride, 55°C, 1h, 9% yield

Precursor **3a** was therefore obtained in four steps starting from the commercially available 5-fluoro-2-hydroxybenzaldehyde based on the publication by Hao et al. [12]. Compound **4** was prepared by a Strecker reaction of 5-fluoro-2-hydroxybenzaldehyde followed by hydrolysis of the resulting cyano group (Scheme 3). 5-Fluoro-2-hydroxybenzaldehyde was dissolved in an ammonia-methanol solution, creating the iminium intermediate, followed by trimethylsilyl cyanide addition, creating the aminonitrile. This was subsequently hydrolyzed using hydrochloric acid in water to afford compound **4**. The newly created amine was boc-protected using di-*tert*-butyl dicarbonate in *tert*-butyl alcohol and sodium hydroxide solution resulting in compound **5**. Amide condensation with 2-aminothiazole, using *N,N*-diisopropylethylamine and propylphosphonic anhydride in ethyl acetate and dimethylformamide gave compound **6**. It was subsequently deprotected using trifluoroacetic acid in dichloromethane before the addition of the aryl halide moiety to the structure by reductive animation. Finally, compound **3a**, was obtained by reacting deprotected **6** with 2-iodobenzaldehyde in methanol, with the pH adjusted by 1,8-diazabicyclo[5.4.0]undec-7-ene (DBU), followed by the addition of the reducing agent sodium cyanoborohydride. The water-soluble nature of the intermediates made isolation challenging and the overall isolated yield varied between syntheses.

### Radiochemistry

**Scheme 4:**
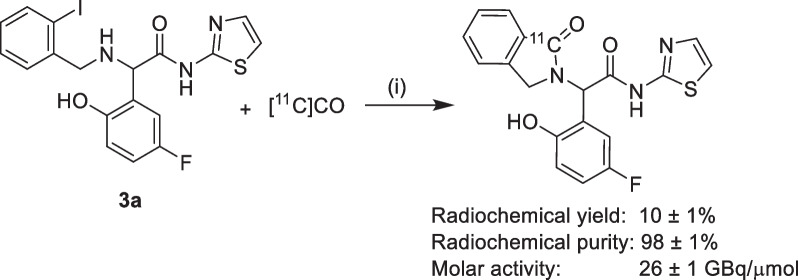
Radiolabeling of EAI045; (i) Tris(dibenzylideneacetone)dipalladium(0), 1,1′-bis(diphenylphosphino)ferrocene, tetrahydrofuran, dimethyl sulfoxide, 100 °C, 10 min

EAI045 was radiolabeled by [^11^C]CO carbonylation, as depicted in Scheme 4. The optimal catalyst was tris(dibenzylideneacetone)-dipalladium, and the addition of 1,1′-bis(diphenylphosphino)ferrocene was vital for the commencement of the reaction. It was imperative to use dry tetrahydrofuran without a stabilizer for the reaction. By adding various solvents like dimethylsulfoxide, dimethylformamide or dimethylacetamide to the tetrahydrofuran, byproduct formation could be avoided. The optimal ratio between tetrahydrofuran and dimethyl sulfoxide was 2:1. [^11^C]CO was transferred to the reaction vial using the xenon transfer method reported by Eriksson et al. [13]. The mixture was heated to 100 °C for 10 min. The product was purified by semi-preparative HPLC, before reformulation in 10% ethanol containing saline solution. An intravenously injectable solution of [^11^C]EAI045 was obtained in a radiochemical yield of 10 ± 1% (decay corrected to end of [^11^C]CO_2_ production), with a molar activity of 26 ± 1 GBq/µmol and purity of ≥ 97% in 51 ± 1 min.

### In vitro evaluation

[^3^H]EAI045 was evaluated in vitro in three human NSCLC cell lines; A549 (control cell line expressing wild-type EGFR), H1975 (expressing the L858R/T790M EGFR mutation), and HCC827 (expressing the del19 EGFR mutation). HCC827 was included in the experiment as Jia et al. hypothesized the del19 EGFR mutation to prevent the opening of the allosteric pocket, rendering EAI045 unable to access it, and demonstrated the lack of inhibition of proliferation in vitro in del19/T790M mutated Ba/F3 cells [7]. This hypothesis was further supported by molecular dynamics simulations by Kannan et al. [14].

**Fig. 1:**
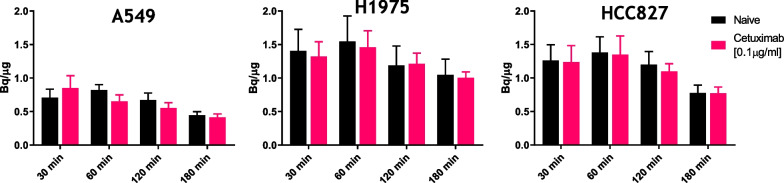
Significant variation in uptake of [^3^H]EAI045 over time (n = 3/time point) was observed in control cell line A549 vs. H1975 and HCC827 cell lines in vitro (P < 0.006)

The uptake of [^3^H]EAI045 varied significantly for all three cell lines over time. Following the peak uptake at 60 min of incubation, the concentration of [^3^H]EAI035 decreased for all cell lines. This was independent of the presence of cetuximab (Fig. 1). The uptake in the EGFR mutated cell lines was significantly higher than in the control cell line (A549 vs. H1975 *P* = 0.0020, A549 vs. HCC827 *P* = 0.0019).

**Fig. 2:**
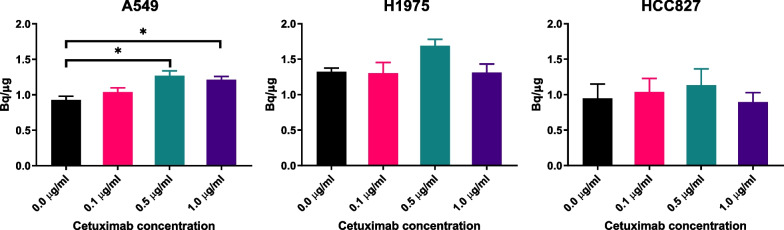
[^3^H]EAI045 uptake as dependent on cetuximab concentration in A549, H1975 and HCC827 (n = 3/concentration) in vitro

The effect of cetuximab concentration on the uptake of [^3^H]EAI045 was investigated in the cell lines. In the A549 cell line, a significant dose-dependent increase in [^3^H]EAI045 uptake was observed (*P* = 0.0436 for 0.5 and 1.0 µg/mL cetuximab, Fig. 2). No other statistically significant changes were observed with cetuximab in the other cell lines. However, the same trend in dose-dependent uptake was observed.

**Fig. 3:**
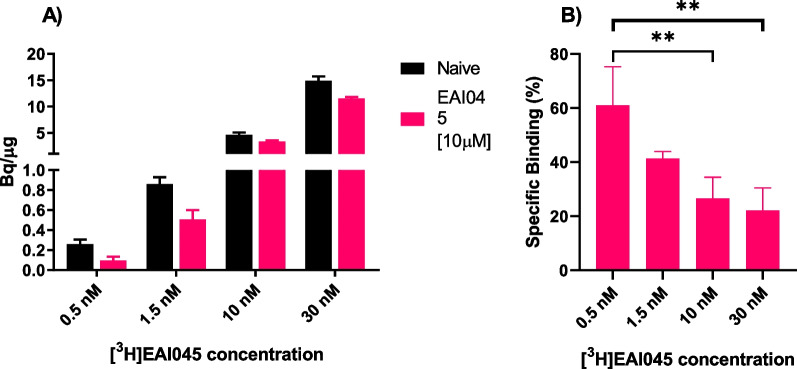
**A** [^3^H]EAI045 uptake in H1975 cells was shown to be concentration dependent. Specific binding of [^3^H]EAI045 uptake in H1975 cell was determined by co-incubating with 10 µM non-labeled EAI045 (n = 3/data point). **B** The specific binding was shown to be significantly higher at lower [^3^H]EAI045 concentrations

Lastly, the concentration dependency of [^3^H]EAI045 on uptake was examined. EAI045 was reported to inhibit EGFR phosphorylation in H1975 cells with a half-maximal effective concentration (EC_50_) of 2 nM [7]. In the current study, adding up to 30 nM [^3^H]EAI045 gave a linear response in the uptake in the H1975 cell line, indicating the maximal uptake to not have been achieved even at 30 nM [^3^H]EAI045. When the tracer-containing medium was supplemented with 10 μM EAI045, a 22–61% reduction in uptake could be observed, however, the [^3^H]EAI045 tracer concentration still indicated a linear uptake in the H1975 cells (Fig. 3).

### In vivo evaluation

#### Metabolite analysis

**Fig. 4:**
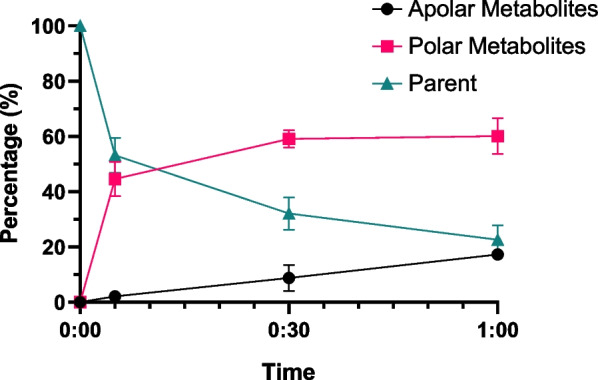
Metabolism of [^11^C]EAI045 in female nu/nu mice bearing A549 xenografts (n = 4 per time point)

The metabolic stability assessment of [^11^C]EAI045 in vivo was carried out in female nu/nu mice bearing A549 xenografts. Following an injection of 10 ± 4 MBq [^11^C]EAI045, the mice were sacrificed at 5-, 30-, and 60-min post-injection (p.i.). At 60 min p.i., 23 ± 5% of the circulating radioactivity in plasma could be attributed to intact [^11^C]EAI045. At this time point, 17 ± 2% was attributed to apolar metabolites, while the majority of the circulating radioactivity originated from polar metabolites (60 ± 6%, Fig. 4). Figure 4 shows a sharp increase in the fraction of polar metabolites during the first five minutes before stabilizing within 30 min p.i. The increase in apolar metabolites is more linear with time.

#### Ex vivo biodistribution in female nu/nu xenografted mice

**Fig. 5:**
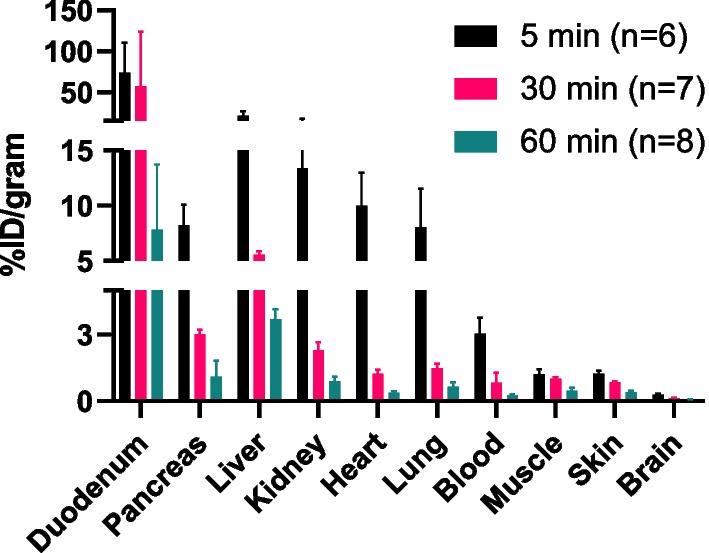
Biodistribution of [^11^C]EAI045 in female nu/nu mice (n = 6 at 5 min, n = 7 at 30 min and n = 8 at 60 min p.i.)

The biodistribution of [^11^C]EAI045 was evaluated at 5-, 30- and 60-min p.i. in female nu/nu mice bearing tumors (Fig. 5). High activity concentrations were measured in the liver (21.7 ± 5.2%ID/g) and kidneys (13.4 ± 4.3%ID/g) in the first five minutes, but by 60 min p.i., the concentration had reduced almost ten-fold (3.7 ± 0.4 and 0.9 ± 0.2%ID/g respectively). Fast clearance of the tracer was observed in the heart, pancreas, blood, and lungs. Very high uptake was detected in the duodenum during the first 30 min (73.7 ± 36.9 at 5 min, 57.3 ± 66.8 at 30 min). The activity in urine increased to 2.3 ± 1.6%ID during the 60 min p.i.

#### Tumor uptake in vivo

**Fig. 6:**
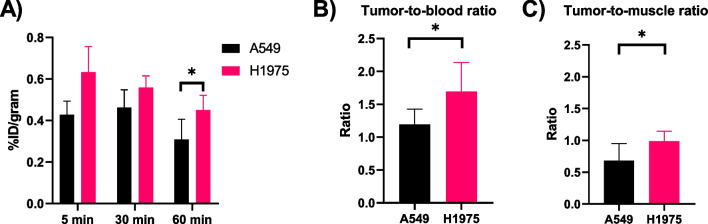
**a** Tumor uptake and retention comparison of [^11^C]EAI045 in A549 (n = 8/time point) and H1975 (n = 4 at 5 min, n = 6 at 30 min, and n = 8 at 60 min p.i.) xenografted mice. **b** Tumor-to-blood ratio and **c** tumor-to-muscle ratio at 60 min p.i. comparison of [^11^C]EAI045 in H1975 (N = 4, n = 8) and A549 (N = 4, n = 8) xenografted mice

The uptake in H1975 xenografts expressing the L858R/T790M mutation was compared to that of A549, which expresses wild-type EGFR (Fig. 6). The activity concentration decreased in both xenografts over time. A statistically significantly higher uptake was observed in H1975 compared to A549 (0.45 ± 0.07%ID/g vs. 0.31 ± 0.10%ID/g, *P* = 0.0166) at 60 min p.i. A similar trend was observed when the activity concentration in the tumors was corrected for blood content (1.70 ± 0 0.44 vs. 1.19 ± 0.23, *P* = 0.0162) or muscle (0.99 ± 0.16 vs. 0.68 ± 0.27, *P* = 0.0176) at 60 min p.i.

#### Influence of cetuximab on biodistribution of [^11^C]EAI045

**Fig. 7:**
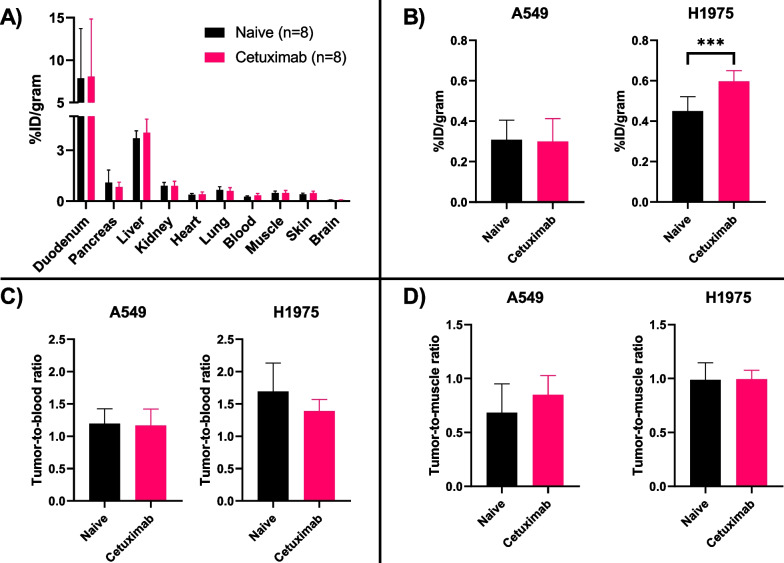
The influence of cetuximab pretreatment on [^11^C]EAI045 **a** biodistribution in xenografted mice (N = 16, n = 8/treatment), **b** tumor uptake, **c** tumor-to-blood ratio, and **d** tumor-to-muscle ratio in A549 (N = 8) and H1975 (N = 8) xenografted (n = 8/treatment) mice at 60 min p.i

The influence of cetuximab on the biodistribution and the A549 or H1975 tumor uptake of [^11^C]EAI045 was assessed by injecting the mice with 0.5 mg cetuximab 24 h prior to tracer injection and the ex vivo biodistribution. As illustrated in Fig. 7a, the pretreatment did not significantly alter the biodistribution of [^11^C]EAI045. Although the uptake in the H1975 tumors seemed higher when pretreated (Fig. 7b, 0.60 ± 0.05%ID/g vs. 0.45 ± 0.07%ID/g), once corrected for blood or muscle radioactivity concentration, no significant difference could be detected (Fig. 7c and d).

## Discussion

Previously Jia et al. demonstrated EAI045 to have a 1000-fold selectivity for L858R/T790M mutated EGFR kinase over wild-type EGFR kinase. They also reported increased inhibitory potency against L858R/T790M EGFR mutated murine cells when cetuximab was used with EAI045. This synergistic effect was assumed to be due to cetuximab's ability to inhibit EGFR dimerization, which would increase the accessibility of the allosteric pocket that EAI045 targets [7].

To further investigate the interaction between EAI045 and EGFR, the compound was labeled with tritium, and the accumulation of the compound was evaluated in three NSCLC cell lines expressing different EGFR mutational statuses. A549, a wild-type EGFR-expressing NSCLC cell line, was used as a negative control. H1975, the cell line Jia et al. used to evaluate the ability of EAI045 to decrease autophosphorylation in L858R/T790M mutated cells [7] and HCC827, an NSCLC cell line expressing the del19 mutation, was used.

Increased uptake of [^3^H]EAI045 was observed in H1975 cells compared to the control cell line A549 in the in vitro uptake assay, indicating selectivity toward the L858R/T790M EGFR mutation. Furthermore, the specific binding window in H1975 cells, as determined using unlabeled EAI045 reached > 60% specific binding at the lowest concentration of [^3^H]EAI045 tested. This suggests the ligand may have high affinity (sub-nanomolar) towards the EGFR mutation and that the cold mass of the radiotracer causes a homologous blocking effect and contributes to a reduction in the specific binding window at higher concentrations. As no other structurally unrelated allosteric EGFR inhibitor is currently available on the market, the selectivity of the [^3^H]EAI045 to the EGFR mutation cannot as yet be confirmed. The uptake of [^3^H]EAI045 in HCC827 cells was also observed to be increased compared to the control cell line A549, contrary to the expectation that the del19 EGFR mutation makes the allosteric pocket inaccessible.

Based on the results from the in vitro cell experiments, the postulated synergetic effect of cetuximab on [^3^H]EAI045 uptake could not be confirmed. Even when the concentration of cetuximab was increased, no significant effect was observed for the EGFR-mutated cell lines. Since we only measured cellular uptake as a measure for binding of [^3^H]EAI045, it cannot be ruled out that cell uptake processes play a role in the total uptake of [^3^H]EAI045 and influences the experimental outcome. Future studies are required to understand the influence cetuximab has on EGFR and how the resulting target modulation influences the binding of allosteric tyrosine kinase inhibitors.

The current study evaluated the metabolism and excretion of [^11^C]EAI045 in mice. Results suggest a rapid metabolism and renal excretion, similar to the findings of Lv et al. in rats [15], as radioactivity in urine increased over time, and plasma metabolite analysis showed up to 60% polar metabolite at 60 min p.i. Fast clearance of radioactivity was observed in most organs, and as lung tissue would be the background tissue for any NSCLC tumors, its fast clearance was important. Skin, a tissue rich in EGFR, had a significantly lower uptake of [^11^C]EAI045 compared to the duodenum, which most likely indicates off-target interaction [16]. The tumor clearance was slower, indicating some interaction/retention with EGFR. The uptake was statistically significantly higher in the L858R/T790M mutated H1975 xenografts than the wild-type EGFR expressing A549, indicating a selective uptake of [^11^C]EAI045 in mutated EGFR xenografts.

In vivo, no significant increase in [^11^C]EAI045 uptake in H1975 xenografts was observed when the mice were pretreated with cetuximab. Consequently, also our in vivo results cannot confirm the hypothesis of synergy between EAI045 and cetuximab from a tumor uptake perspective. However, it cannot be excluded that this is due to confounding observations like a high level of off-target binding or apolar metabolite interaction.

## Conclusions

EAI045 was successfully labeled with tritium and carbon-11. Both in vivo ([^11^C]EAI045) and in vitro ([^3^H]EAI045), a higher uptake was achieved in L858R/T790M mutated EGFR expressing H1975 compared to wild-type EGFR expressing A549. We could not confirm the theory on the interaction of EAI045 and cetuximab as hypothesized by Jia et al.

## Methods

### General

Chemicals and solvents were obtained from commercial sources (Merck/Sigma Aldrich (Darmstadt, Germany), Fluorochem (Hadfield, United Kingdom), Fisher Scientific (Landsmeer, the Netherlands), Biosolve (Valkenswaard, the Netherlands), Axon Medchem (Groningen, the Netherlands), and Selleck Chemicals (Houston, TX, USA)) and used without further purification. NMR spectra were recorded on Bruker AVANCE II 500 (500 MHz for ^1^H and 550 MHz for ^3^H), Bruker AVANCE II 500 Ascend (500 MHz for ^1^H, 126 MHz for ^13^C), or Bruker AVANCE III HD 600 (600MHz for ^1^H) at 20°C. Chemical shifts (δ) are reported in parts per million (ppm) relative to the solvent (dimethyl sulfoxide (DMSO)-d_6_, ^1^H 2.50 ppm, ^13^C 39.52 ppm, methanol (MeOD-d_4_), ^1^H 3.31 ppm, ^13^C 49.00 ppm). Coupling constants (*J*) are reported in units of hertz (Hz). The following abbreviations are used to describe multiplicities: s (singlet), d (doublet), t (triplet), q (quartet), quin (quintet), m (multiplet), br (broad). High-resolution mass spectra (HRMS, m/z) analyses were conducted on a Bruker microQTOF MS apparatus (Capillary voltage: − 4500 V; collision energy: 5 eV) using positive (ESI+) electrospray ionization. Thin-layer chromatography (TLC) was performed using TLC plates from Merck (aluminum TLC plates, silica gel coated with fluorescent indicator F254). Compounds on the TLC plate were visualized by UV light at 254 nm or by general TLC staining procedures if required. Using silica-packed cartridges, flash column chromatography was performed on a Büchi Sepacore® X10 flash system. Aldrich silica gel 60A (230–400 mesh) was used for preparing pre-column cartridges.

### Synthesis

#### 2-(5-fluoro-2-hydroxyphenyl)-*N*-(5-iodothiazol-2-yl)-2-(1-oxoisoindolin-2-yl)acetamide

EAI045 (50 mg, 0.12 mmol) and *N*-iodosuccimide (29 mg, 0.13 mmol) were dissolved in dichloromethane (5 mL) containing 10% trifluoroacetic acid (%v/v). The reaction mixture was vigorously stirred at room temperature for 2.5 h. High-performance liquid chromatography (HPLC) was used to monitor the progression of the reaction (Alltima C18, 250 × 4.6 mm, 5 µm, 60% acetonitrile in water). Due to high insolubility, the product was purified on an “as needed” basis using preparative HPLC (Alltima C18, 250 × 10 mm, 5 µm, 70% acetonitrile in water, 5 mL·min^−1^). Therefore, no yield is available. Acquiring a satisfactory ^13^C-NMR proved challenging due to the low solubility, so the structure was confirmed by comparing the ^1^H-NMR to EAI045. The proton which was not present in the ^1^H-NMR for 2-(5-fluoro-2-hydroxyphenyl)-*N*-(5-iodothiazol-2-yl)-2-(1-oxoisoindolin-2-yl)acetamide compared to EAI045, was confirmed to have been replaced by iodine by HRMS.

^1^H NMR (600 MHz, MeOD-*d4*): d = 7.82 (d, *J* = 7.7 Hz, 1H), 7.61 (td, *J* = 7.7, 1.1 Hz, 1H), 7.50–7.54 (m, 2H), 7.47 (s, 1H), 7.03 (td, *J* = 8.4, 2.9 Hz, 1H), 6.96 (dd, *J* = 9.2, 2.9 Hz, 1H), 6.88 (dd, *J* = 9.2, 4.8 Hz, 1H), 6.45 (s, 1H), 4.77 (d, *J* = 17.2 Hz, 1H), 4.05 (d, *J* = 17.2 Hz, 1H).

HRMS (ESI + , m/z) calc. for C_13_H_17_FNO_5_ 509.9779 (M + H) found 509.9754.

#### [^3^H]EAI045

Reactions with tritium gas (RC Tritec) were performed on an RC Tritec tritium manifold. Analytical HPLC was performed using a Waters 2695 solvent handling system with a Packard Radiomatic 500TR Flow Scintillation Analyzer in-line radioactivity detector (after UV detection), while semi-preparative HPLC was performed using a system composed of a Gilson 322 Pump equipped with a Gilson UV/VIS‐152 detector. For the analytical HPLC, a Waters XBridge C18 (3.5 μm, 4.6 × 100 mm, at 60 °C) column was used, and a gradient elution method was employed (0–3 min: 5% B; 3–25 min: 5–95% B; 25–30 min: 95% B, 0.6 mL∙min^−1^, UV 254 nm, where A = 10 mMol ammonium formate (pH 3) and B = acetonitrile, example of a typical HPLC can be found in Additional file 1: Supplemental information).

LCMS was acquired on a Waters Acquity UPLC using a BEH C18 column (1.7 μm, 50 mm × 2.1 mm) with a gradient method (0–0.2 min: 10% B; 0.2–1.7 min: 99% B; 1.7–1.8 min: 99% B; 1.80–1.81 min: 10% B, 1 mL∙min^−1^, at 60°C where A = 10 mMol ammonium formate (pH 10) and B = acetonitrile) and electrospray ionization.

2-(5-fluoro-2-hydroxyphenyl)-*N*-(5-iodothiazol-2-yl)-2-(1-oxoisoindolin-2-yl)acetamide (1.90 mg, 3.73 µmol) was added to a slurry of palladium on calcium carbonate (10% Pd, 2.80 mg, 0.003 mmol) and ethanol (absolute, 99.5%, 0.5 mL). The mixture was de-gassed by three freeze–thaw cycles. Gaseous tritium (453 GBq, 0.43 mmol) was introduced to the flask, and the mixture was stirred at ambient temperature for 4 h. Unreacted tritium was recovered, volatiles were removed by a stream of nitrogen, methanol was added and removed by a stream of nitrogen twice. The residue was suspended in methanol, and the flask was rinsed with 15 mL methanol. The combined methanol mixture was filtered and concentrated, yielding around 1.6 GBq of crude product. The solid was taken up in dimethyl sulfoxide and was purified by reverse-phase chromatography (XBridge Prep C18 5μ OBD 19 × 100 mm, 10–65% acetonitrile in water containing 0.1% trifluoroacetic acid, 25 min, flow rate 10 mL∙min^−1^, λ 230 nm, fc level 75). Tritium was lost from the compound slowly (approx. 2%/week). Due to this, a second purification was required (XBridge Prep C18 5μ OBD 19 × 250 mm, 20–65% acetonitrile in water (both sparged with helium), 25 min, flow rate 10 mL∙min^−1^, λ 230 nm, fc level 50 MQ water). This yielded 730 MBq of [^3^H]EAI045 in a radiochemical yield of 0.2%, a radiochemical purity of 98%, and a molar activity of 763 kBq/nmol. It was stored in helium-sparged ethanol under nitrogen at -80°C.

^1^H NMR (500 MHz, MeOD-*d4*) δ 7.84 (d, *J* = 7.5 Hz, 1H), 7.63 (t, *J* = 7.4 Hz, 1H), 7.54 (t, *J* = 8.7 Hz, 1H), 7.52 (m, 1H), 7.45 (s, 1H), 7.19 (m, 0.3 H), 7.05 (td, *J* = 8.8, 2.2, Hz, 1 H), 6.99 (d, *J* = 9.1 Hz, 1H), 6.91 (dd, *J* = 8.9, 4.5 Hz, 1H), 6.49 (s, 1H), 4.07 (d, *J* = 17.4 Hz, 2H). The spectrum shows considerable residual ethanol from the storage solution.

^3^H NMR (550 MHz, MeOD-*d4*): 7.22 (93%), 6.94 (d, J = 4.5 Hz, 7%).

LC–MS (M + 1 (%)): 384.1 (34%), 386.4 (100%), 388.3 (13%).

#### [^11^C]EAI045 precursor synthesis

*2-amino-2-(5-fluoro-2-hydroxyphenyl)acetic acid,* ***4***

 5-fluoro-2-hydroxybenzaldehyde (10.0 g, 71.4 mmol) was dissolved in an ammonia solution (7N in methanol, 200 mL). The solution was cooled to 0°C before the dropwise addition of trimethylsilyl cyanide (13.50 mL, 107.9 mmol), and the resulting mixture was stirred for 10 min at that temperature. Hereafter, the reaction mixture was heated to 45°C and stirred vigorously until TLC showed no more starting material (60 min). The reaction mixture was concentrated before hydrochloric acid (2 M, 100 mL) and ethyl acetate (100 mL) were added. The phases were separated, and the aqueous phase was washed with ethyl acetate (3 × 50 mL). Concentrated hydrochloric acid was added (50 mL) to the aqueous phase, and the reaction mixture was refluxed for two hours. Activated carbon (2.5 g) was added to the reaction mixture, and the reaction mixture was heated to reflux for 30 min. The hot reaction mixture was filtered over a pad of celite and concentrated on the rotary evaporator yielding **4** that was used for the next step without further purification.

^1^H NMR (500 MHz, MeOD-*d4*) δ 7.11 (dd, J = 8.7, 3.1 Hz, 1H), 7.05 (td, J = 8.5, 3.0 Hz, 1H), 6.91 (dd, J = 8.9, 4.5 Hz, 1H), 5.15 (s, 1H).

^13^C NMR (126 MHz, MeOD-*d4*) δ 170.59, 157.41 (d, *J* = 236.7 Hz), 153.20 (d, *J* = 2.2 Hz), 121.44 (d, *J* = 6.5 Hz), 118.57 (d, *J* = 23.2 Hz), 117.60 (2C, dd, *J* = 16.2, 8.3 Hz), 53.86.

HRMS (ESI + , m/z) calc. for C_8_H_9_FNO_3_ 186.0561 (M + H) found 186.0555.

*2-((tert-butoxycarbonyl)amino)-2-(5-fluoro-2-hydroxyphenyl)acetic acid,* ***5***

 As obtaining a correct weight for compound **4** was not possible due to the salts still present, the reagents in this step were calculated to a theoretical total conversion of 5-fluoro-2-hydroxybenzaldehyde to compound **4**. *Tert*-butanol (40 mL) was added to the flask described above containing compound **4**. The pH was adjusted with a sodium hydroxide solution (2 M) until pH 12 was reached. Subsequently, di-*tert*-butyl dicarbonate (17.13 g, 78.5 mmol) was added to the solution. The resulting reaction mixture was stirred overnight at room temperature and TLC determined the completion of the reaction. Ethyl acetate (200 mL) and water (200 mL) were added to the reaction mixture. Phases were separated, and the aqueous phase was washed with ethyl acetate (2 × 50 mL), before cautiously adjusting the pH to pH 5 with a hydrochloric acid solution (2 M). The aqueous phase was extracted with ethyl acetate (3 × 50 mL), and the combined organic phases were dried over Na_2_SO_4_ and concentrated. A brown residue was obtained (0.93 g, 5% over two steps) and was used for the next step without further purification.

^1^H NMR (500 MHz, MeOD-*d4*) δ 6.96 (dd, *J* = 9.2, 3.1 Hz, 1H), 6.86 (td, *J* = 8.5, 3.1 Hz, 1H), 6.78 (dd, *J* = 8.8, 4.6 Hz, 1H), 5.37 (s, 1H), 1.44 (s, 9H).

^13^C NMR (126 MHz, MeOD-*d4*) δ 174.81, 157.67 (d, *J* = 235.4 Hz), 152.60 (d, *J* = 2.3 Hz), 127.50 (d, *J* = 7.3 Hz), 117.63 (d, *J* = 7.7 Hz), 116.00 (d, *J* = 23.1 Hz), 115.75 (d, *J* = 24.3 Hz), 80.79, 55.00, 28.64.

HRMS (ESI + , m/z) calc. for C_13_H_17_FNO_5_ 286.1085 (M + H) found 286.1073.

*tert-butyl (tert-butoxycarbonyl)(1-(5-fluoro-2-hydroxyphenyl)-2-oxo-2-(thiazol-2-ylamino)ethyl)carbamate,* ***6***

Compound **5** (0.93 g, 3.26 mmol) was dissolved in ethyl acetate (7 mL) and dimethylformamide (0.85 mL). 2-aminothiazole (0.33 g, 3.3 mmol) along with *N,N*-diisopropylethylamine (1.70 mL, 86.1 mmol) were added to the mixture, followed by the dropwise addition of propyl phosphonic anhydride (50% ethyl acetate, 3 mL, 5.0 mmol). The reaction mixture was stirred at room temperature overnight. Hereafter, the reaction mixture was quenched by adding saturated sodium bicarbonate solution (30 mL) and stirred for 30 min. The aqueous phase was extracted with ethyl acetate (3 × 20 mL). The combined organic phases were washed with water, dried over sodium sulfate_,_ and concentrated. The crude product was purified by flash column chromatography (3–10% methanol in dichloromethane). Fractions containing the product were combined and concentrated, yielding a yellow solid (180 mg, 0.5 mmol, 15% yield).

^1^H NMR (500 MHz, MeOD-*d4*) δ 7.41 (d, *J* = 3.6 Hz, 1H), 7.12 (d, *J* = 3.6 Hz, 1H), 7.05 (dd, *J* = 9.2, 3.1 Hz, 1H), 6.92 (td, *J* = 8.5, 3.1 Hz, 1H), 6.84 (dd, *J* = 8.9, 4.6 Hz, 1H), 5.72 (s, 1H), 1.47 (s, 9H).

^13^C NMR (126 MHz, MeOD-*d4*) δ 169.27, 164.21, 157.47 (d, *J* = 236.0 Hz), 152.47, 137.93, 124.95, 117.22 (d, *J* = 7.7 Hz), 116.53 (d, *J* = 23.8 Hz), 108.24, 53.33, 28.66.

HRMS (ESI + , m/z) calc. for C_13_H_17_FNO_5_ 368.1075 (M + H) found 368.1026.

*2-(5-fluoro-2-hydroxyphenyl)-2-((2-iodobenzyl)amino)-N-(thiazol-2-yl)acetamide,* ***3a.***

Compound **6** (180 mg, 0.5 mmol) was suspended in dichloromethane (2 mL). Trifluoroacetic acid (1.9 mL, 24.8 mmol) was added dropwise, and the reaction mixture was stirred for 15 min before being concentrated on a rotary evaporator. Dry methanol (10 mL) was added to the residue and 1,8-diazabicyclo(5.4.0)undec-7-ene was used to adjust the pH from pH 1 to pH 5. Sodium sulfate (146 mg, 1.0 mmol) was added to the reaction mixture, followed by 2-iodobenzaldehyde (114 mg, 0.5 mmol). The reaction mixture was heated to 60°C for 1.5 h (the absence of starting material was confirmed by TLC). Sodium cyanoborohydride (154 mg, 2.5 mmol) was added batch-wise to the reaction mixture. The reaction mixture was stirred at 60°C for three hours, after which it was stirred at room temperature overnight. After concentration in vacuo, water (10 mL) and dichloromethane (10 mL) were added. Phases were separated, and the aqueous phase was extracted with dichloromethane (2 × 10 mL). The combined organic phases were washed with brine, dried over Na_2_SO_4_, and concentrated. The crude product was purified by flash column chromatography (1% methanol in dichloromethane). Fractions containing the product were combined and concentrated. The product was further purified on semi-preparative HPLC (Alltima 5 µm C18 250 × 10 mm column, 55% acetonitrile in water), yielding a colorless solid (21.6 mg, 0.1 mmol) in 9% yield.

^1^H NMR (500 MHz, MeOD-*d4*) δ 7.82 (dd, *J* = 7.9, 1.2 Hz, 1H), 7.47 (dd, *J* = 7.6, 1.7 Hz, 1H), 7.43 (d, *J* = 3.6 Hz, 1H), 7.36 (td, *J* = 7.5, 1.2 Hz, 1H), 7.13 (d, *J* = 3.6 Hz, 1H), 7.02–6.96 (m, 2H), 6.90 (td, *J* = 8.5, 3.1 Hz, 1H), 6.79 (dd, *J* = 8.8, 4.6 Hz, 1H), 4.66 (s, 1H), 3.93–3.84 (m, 2H).

^13^C NMR (126 MHz, MeOD-*d4*) δ 172.22, 159.71, 157.65 (d, *J* = 236.6 Hz), 152.85 (d, *J* = 2.2 Hz), 142.55, 140.77, 138.28, 131.30, 130.30, 129.60, 126.68 (d, *J* = 6.5 Hz), 117.38 (d, *J* = 7.8 Hz), 117.06 (d, *J* = 24.1 Hz), 116.46 (d, *J* = 23.0 Hz), 114.91, 100.47, 62.63, 57.14.

HRMS (ESI + , m/z) calc. for C_18_H_16_FIN_3_O_2_S 483.9986 (M + H) found 484.0039.

#### [^11^C]EAI045

Chemicals and solvents were obtained from commercial sources and used as received. Dry tetrahydrofuran without stabilizing agents (such as butylated hydroxytoluene) was used whenever tetrahydrofuran was mentioned. EAI045 was purchased from Selleck Chemicals (Houston, TX, USA). Semi-preparative isocratic high-performance liquid chromatography (HPLC) was performed using a Jasco PU-1587 station with a Jasco UV1575 UV detector (254 nm), a custom-made radioactivity detector, and chromatograms were acquired using Jasco ChromNAV CFR software (version 1.14.01).

The radiochemical purity and molar activity were determined by analytical HPLC, using a Shimadzu SPD20A system with either a Raytest 2 × 2 pinhole NaI-Detector (Straubenhardt, Germany) or a Scionix Holland VD14-E1 (Bunnik, The Netherlands) detector and LabSolutions 5.85 software (Shimadzu Corporation, Japan). Quality control of the final formulated product was performed by analytical HPLC using an Alltima C18 5 µm 250 × 4.6 mm column with an eluent gradient of; 0–3 min: 40% B; 3–16 min: 40–100% B; 16–18 min: 100% B, 18–23 min: 100–40% B, 1 mL∙min^−1^, UV 254 nm, where A = water containing 0.1% trifluoroacetic acid and B = acetonitrile containing 0.1% trifluoroacetic acid (example of a typical HPLC can be found in Additional file 1: Supplemental information). Radioactivity levels were measured using a Veenstra VDC-405 dose calibrator (Joure, The Netherlands). Radiochemistry was carried out in homemade, remotely controlled synthesis units. Radiochemical yields and molar activity were defined following the recently published radiochemistry nomenclature guideline [17].

The [^11^C]CO used for the labeling [^11^C]EAI045 was produced from [^11^C]CO_2_. The [^11^C]CO_2_ was produced by a ^14^N(p,α)^11^C nuclear reaction performed in a 0.5% O_2_/N_2_ gas mixture using an IBA Cyclone 18/9 cyclotron (IBA, Louvain-la-Neuve, Belgium). Subsequently, the [^11^C]CO_2_ was transferred to an in-house built synthesis unit, previously described [13]. The [^11^C]CO_2_ was directed over a gas purifier column (400 × 4 mm, silica gel, 100/80 mesh) using helium (15 mL∙min^−1^) as carrier gas. The purified [^11^C]CO_2_ was subsequently passed over a molybdenum column (Sigma Aldrich, 150μm, 99.99%) heated to 850 °C after which any unreacted [^11^C]CO_2_ was trapped on an ascarite® column, and [^11^C]CO was collected on a silica trap (− 150°C, 1 mg silica gel, 100/80 mesh). Finally, the trap was heated to 20°C to allow transfer of the [^11^C]CO to the reaction vial using a 3.0 mL∙min^−1^ flow of xenon (Fluka, ≥ 99.995).

[^11^C]EAI045 was synthesized using [^11^C]CO generated as presented above. The precursor (compound **4**, 2.4 mg, 5.0 μmol) was dissolved in 50 μL dry tetrahydrofuran without a stabilizer. Tris(dibenzylideneacetone)dipalladium (2.0–2.7 mg, 2.2–2.9 μmol) was added along with 50 μL dry dimethyl sulfoxide. After 1 h at room temperature, 1,1′-bis(diphenylphosphino)ferrocene (2.7 mg, 4.9 μmol) dissolved in 50 μL tetrahydrofuran was added to the mixture and thoroughly mixed. 400 μL tetrahydrofuran and 200 μL dimethyl sulfoxide was added before capping the 0.9 mL vial. [^11^C]CO in xenon gas was introduced to the reaction mixture. The needle was removed, and the vial was fitted with a pressure cap. The reaction mixture was heated to 100 °C for 10 min. The pressure cap was removed after the reaction mixture was cooled to 30 °C, and the reaction mixture was transferred to a reservoir containing 0.75 mL HPLC eluent. The diluted fraction was filtered before semi-preparative HPLC purification using an Alltima 5 µm C18 250 × 10 mm column and using 42% acetonitrile in water with 0.1% trifluoroacetic acid added as eluent at a flow rate of 5 mLmin^−1^. The collected fraction was diluted with 30 mL water and passed over a tC18 Sep-Pak Light cartridge (pre-conditioned with 10 mL ethanol and 10 mL water). After washing the cartridge with 15 mL of water, [^11^C]EAI045 was eluted with 0.5 mL sterile ethanol and diluted with 4.5 mL of saline, resulting in a 10% ethanol (%v/v) containing intravenously injectable solution.

### In vitro evaluation

#### Cell culture

Cell lines HCC827 and H1975 (American Type Culture Collection, Manassas, VA) were cultured in RPMI1640 medium supplemented with 10% fetal bovine serum (FBS) and 100 U/mL penicillin–streptomycin. Cell line A549 (American Type Culture Collection, Manassas, VA) was cultured in Ham’s F-12K (Kaighn's) medium supplemented with 10% FBS and 100 U/mL penicillin–streptomycin. Cells were maintained under standard cell culture conditions at 37 °C and 5% CO_2_ in humid conditions.

#### Time-dependent tracer uptake assay

0.11 × ﻿10^6^ (A549/H1975/HCC827) cells were seeded onto disposable 12-well plates (Corning, Costar) to give the desired cell density. The plates were incubated in complete growth medium for one day in the above described conditions (1 mL/well). An hour before the experiment, the medium was replaced with unsupplemented RPMI-1640 medium without phenol red (experiment medium). The uptake was elucidated by adding 1 mL of the experiment medium containing the tracer at a concentration of 1.3 nM. The tracer solution contained 0.1 µg/mL cetuximab when evaluating the influence of cetuximab on the uptake. The plates were incubated at 37 °C in a humid environment for 0.5, 1, 2, or 3 h. The tracer-containing medium was removed, and the cells were washed twice with cold 0.5 mL phosphate-buffered saline (PBS, Gibco). 0.5 mL of 5% sodium dodecyl sulfate (SDS) was added to each well to denature the cells. 0.5 mL of PBS was used to dilute the resulting mixture, and 100 µL was used to determine the activity in the sample using a 2900TR Tricarb beta counter (Perkin Elmer). All samples were diluted 1:1 to determine protein concentration by micro bicinchoninic acid protein assay (µBCA, ThermoFisher). All experiments were performed in triplicate.

#### Cetuximab concentration-dependent tracer uptake assay

0.11 × 10^6^ (A549/H1975/HCC827) cells were seeded onto disposable 12-well plates (Corning, Costar) to give the desired cell density. The plates were incubated in complete growth medium for one day in the described conditions. An hour before the experiment, the medium was replaced with unsupplemented RPMI-1640 medium without phenol red (experiment medium). The added tracer solution contained cetuximab in varying concentrations (0, 0.1, 0.5 or 1 µg/mL). The plates were incubated at 37 °C in a humid environment for one hour. The tracer-containing medium was removed, and the cells were washed twice with cold 0.5 mL PBS (Gibco). 0.5 mL of 5% SDS was added to each well to denature the cells. 0.5 mL of PBS was used to dilute the resulting mixture, and 100 µL was used to determine the activity in the sample. All samples were diluted 1:1 to determine protein concentration by micro bicinchoninic acid protein assay (µBCA, ThermoFisher). All experiments were done in triplicate.

#### Tracer concentration and blocking uptake assay

0.11 × 10^6^ H1975 cells were seeded onto disposable 12-well plates (Corning, Costar) to give the desired cell density. The plates were incubated in complete growth medium for one day in the described conditions. An hour before the experiment, the medium was replaced with unsupplemented RPMI-1640 medium without phenol red (experiment medium).

The tracer solution added to the wells contained varying concentrations of tracer (0.5, 1.5, 10, or 30 nM). This series was repeated, containing 10 µM of cold EAI045. The plates were incubated at 37 °C in a humid environment for one hour. The tracer-containing medium was removed, and cells were washed twice with cold 0.5 mL PBS (Gibco). 0.5 mL of 5% SDS was added to each well to denature the cells. 0.5 mL of PBS was used to dilute the resulting mixture, and 100 µl was used to determine the activity in the sample. All samples were diluted 1:1 to determine protein concentration by micro bicinchoninic acid protein assay (µBCA, ThermoFisher). All experiments were done in triplicate.

### In vivo evaluation

#### Cell lines and reagents

A549 and H1975 were acquired from LCG (ATCC, Wesel, Germany). The human NSCLC cell line A549 was cultured in F-12K Medium (Kaighn's Modification of Ham’s F-12 Medium, Gibco, ThermoFisher Scientific, Waltham, MA, USA) supplemented with 10% FBS (Gibco life technologies, Thermo Fisher Scientific, Waltham, MA, USA). H1975 was cultured in RPMI 1640 medium (ATCC modification, Gibco, ThermoFisher Scientific, Waltham, MA, USA) supplemented with 10% FBS (Gibco, ThermoFisher Scientific, Waltham, MA, USA). The cell lines were maintained under standard cell culture conditions at 37 °C in a water-saturated atmosphere of 5% CO_2_ in the air according to ATCC guidelines.

#### Xenografts

Female athymic Nude-Foxn1nu mice (18 to 28 g, 7 weeks, Envigo, Horst, The Netherlands) were group-housed in pre-sterilized cages under standard conditions (20–24 °C, 40–70% relative humidity, 12-h light/dark cycles) and provided with ad libitum access to sterilized water and irradiated sterilized Teklad mouse food. Animal experiments were performed in accordance with the European Community Council Directive (2010/63/EU) for laboratory animal care and the Dutch Law on animal experimentation. The experimental protocol was validated and approved by the central committee for animal experimentation (CCD) and the local committee on animal experimentation of the VU University Medical Center. Animals were allowed to acclimate for at least one week before the injection of tumor cells. Subcutaneous tumors were induced by injecting a suspension of 2.2–2.5 × 10^6^ A549 or H1975 cells in PBS, in both flanks under isoflurane anesthetics (1–2% in oxygen). Once most tumors reached a suitable size (100 to 200 mm^3^), the mice were used for the studies (3–5 weeks p.i.) for A549, 2–3 weeks for H1975).

#### Metabolic stability study

The metabolic stability of [^11^C]EAI045 was analyzed in female nu/nu mice bearing A549 tumors. The mice were intravenously injected with 10 ± 4 MBq tracer and were sacrificed at 5-, 30-, and 60-min p.i. (n = 4 for each time-point). 0.6–1 mL of blood was collected via heart puncture, and the plasma was separated from the blood cells by centrifugation (4000 rpm for 5 min). Recovered plasma (100–300 µL) was diluted with 2 mL 0.15 M hydrochloric acid before loading onto an activated tC2 Sep-Pak solid-phase extraction (SPE) cartridge. The filtrate was collected together with any remaining polar metabolites that were washed off the cartridge with water. The apolar fraction was collected by eluting the cartridge with a mixture of methanol and water. The collected fractions were counted for radioactivity in a Wizard Gammacounter 2480 (Wallac/PerkinElmer, Waltham, MA, USA). The apolar fraction was analyzed using semi-preparative radio-HPLC on a Dionex Ultimate 3000 system. The percentage of intact tracer was determined using a Phenomenex Gemini (250 × 10 mm, 5 µm) column and acetonitrile in water containing trifluoroacetic acid (0.1%) as eluent (gradient method: 25–60% acetonitrile over 12 min at a flow of 3 mL·min^−1^). Fractions of 30 s were collected and counted for radioactivity in a Wizard Gammacounter 1470 (Wallac/PerkinElmer, Waltham, MA, USA).

#### Ex vivo biodistribution

12 female nu/nu mice bearing A549 tumors and 9 female nu/nu mice bearing H1975 tumors were injected intravenously with 10 ± 4 MBq [^11^C]EAI045 under isoflurane anesthesia (2% in 1 L·min^−1^). The mice were sacrificed at 5-, 30-, 60-min p.i. Lung, kidney, liver, duodenum, pancreas, heart, tail (site of injection), brain, blood muscle, skin, and tumors (left and right) were collected, weighed, and counted for radioactivity in a Wallac Compugamma 1210 counter (PerkinElmer, Turku, Finland). The percentage of the injected dose per gram of tissue (%ID/g) was calculated. Error bars indicate standard deviation (SD).

#### Influence of cetuximab on ex vivo biodistribution

Four female H1975 tumor-bearing and four female A549 tumor-bearing nu/nu mice were injected intravenously with 0.5 mg cetuximab (Merck, Erbitux, 5 mg/mL), 24 h before being injected with 10 ± 4 MBq [^11^C]EAI045 under isoflurane anesthesia (2% in 1 L·min^-1^). The dose and timing of the cetuximab injection was based on a publication by Lee and Tannock who found that at higher doses a relatively uniform distribution can be achieved after about 24 h. [18] The mice were sacrificed 60 min p.i. Lung, kidney, liver, duodenum, pancreas, heart, tail (site of injection), brain, blood muscle, skin, and tumors (left and right) were collected, weighed, and counted for radioactivity in a Wallac Compugamma 1210 counter (PerkinElmer, Turku, Finland). The percentage of the injected dose per gram of tissue (%ID/g) was calculated. Error bars indicate the standard deviation.

### Statistics

All values are expressed as mean ± standard deviation. The results were analyzed using GraphPad Prism 8.0.2 software. A value of *P* < 0.05 was considered significant. For in vitro uptake of [^3^H]EAI045, a repeated measures two-way ANOVA with Geisser–Greenhouse correction was run for each cell line comparing the treatment (naïve or co-incubation with cetuximab) and the uptake over time. A repeated measures two-way ANOVA with the Geisser-Greenhouse correction with a Dunnett’s multiple comparisons test was run to compare the main effect of the uptake in the cell lines. The statistical significance of the cetuximab concentration influence on [^3^H]EAI045 uptake in cells was evaluated using Brown–Forsythe and Welch ANOVA tests, with Holm-Sidak's multiple comparisons test, with individual variances computed for each comparison (Dunnett T3). The significance in difference between specific binding was evaluated using an ordinary one-way ANOVA with a Tukey’s multiple comparisons test with a single pooled variance. Uptake of [^11^C]EAI045 over time in A549 and H1975 xenografts was evaluated for statistical significance using a mixed-effects model with the Geisser/Greenhouse correction, with Sidak’s multiple comparisons test, with individual variances computed for each comparison. The significance of the difference between tumor-to-blood and tumor-to-muscle ratios in A549 and H1975 xenografted mice was evaluated using a two-tailed t-test with Welch's correction. The influence of cetuximab on the [^11^C]EAI045 biodistribution was evaluated for significance using a repeated measures two-way ANOVA and Sidak’s multiple comparisons test, with a single pooled variance. The difference between tumor, tumor-to-blood, and tumor-to-muscle ratio was evaluated for significance using a two-tailed t-test with Welch's correction.

### Supplementary Information

**Additional file 1**. **Figure S1.** Radio-HPLC chromatogram of [^3^H]EAI045. **Figure S2.** An example of a typical radio-HPLC chromatogram for [^11^C]EAI045.

## Data Availability

All data generated or analysed during this study are included in this published article (and its supplementary information file).

## Abbreviations

ATP: Adenosine triphosphate
CCD: Central committee for animal experimentation
EC_50_: Half-maximal effective concentration
EGFR: Epidermal growth factor receptor
HPLC: High performance liquid chromatography
HRMS: High-resolution mass spectrometry
LCMS: Liquid chromatography–mass spectrometry
NMR: Nuclear magnetic resonance spectroscopy
NSCLC: Non-small cell lung cancer
SD: Standard deviation
SDS: Sodium dodecyl sulfate
SPE: Solid-phase extraction
PBS: Phosphate-buffered saline
PET: Positron emission tomography
p.i.: Post-injection
TLC: Thin-layer chromatography
TKI: Tyrosine kinase inhibitor
UV: Ultra violet
%ID/g: Percent of injected dose per gram
