# Supplementary material for: Synthesis and preclinical evaluation of [11C]EAI045 as a PET tracer for imaging tumors expressing mutated epidermal growth factor receptor
Source: EJNMMI Res. 2024 Feb 16;14:19. doi: 10.1186/s13550-024-01078-6 (PMC10873260; doi:10.1186/s13550-024-01078-6)
Supplement: Supplementary file 1 — Additional file 1. Figure S1. Radio-HPLC chromatogram of [3H]EAI045. Figure S2. An example of a typical radio-HPLC chromatogram for [11C]EAI045. [file 13550_2024_1078_MOESM1_ESM.docx]

# Synthesis and preclinical evaluation of [^11^C]EAI045 as a PET tracer for imaging tumors expressing mutated epidermal growth factor receptor

Antonia A. Högnäsbacka^1,2^, Alex J. Poot^1,2^, Christophe Plisson^3^, Jonas Bergare^4^, David R. Bonsall^3^, Stuart P. McCluskey^3^, Lisa A. Wells^3^, Esther Kooijman^1,2^, Robert C. Schuit^1,2^, Mariska Verlaan^1,2^, Wissam Beaino^1,2^, Guus A.M.S. van Dongen^1,2^, Danielle J. Vugts^1,2^, Charles S. Elmore^4^, Jan Passchier^3^, Albert D. Windhorst^1,2^

^1^Amsterdam UMC, Vrije Universiteit Amsterdam, Dept. Radiology & Nuclear Medicine, De Boelelaan 1117, 1081HV, Amsterdam, The Netherlands

^2^Cancer Center Amsterdam, Biomarkers & Imaging, Amsterdam, the Netherlands

^3^Invicro LLC, London W12 0NN, UK

^4^Early Chemical Development, Pharmaceutical Sciences, R&D AstraZeneca, Gothenburg, Sweden


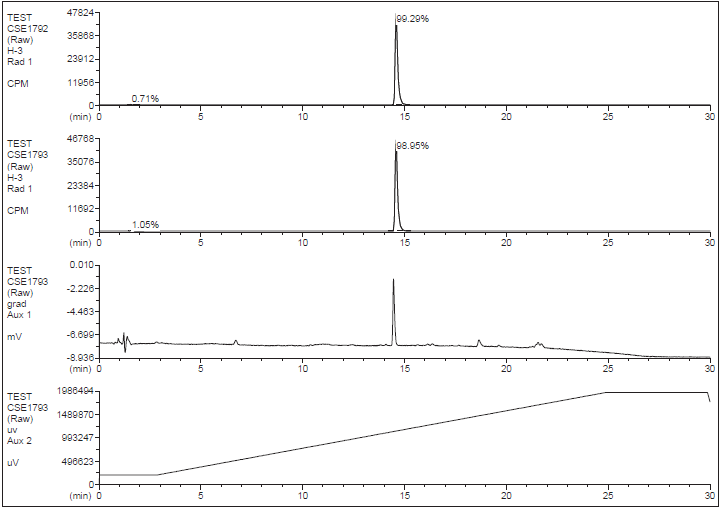


Figure S1. Radio-HPLC chromatogram of [^3^H]EAI045


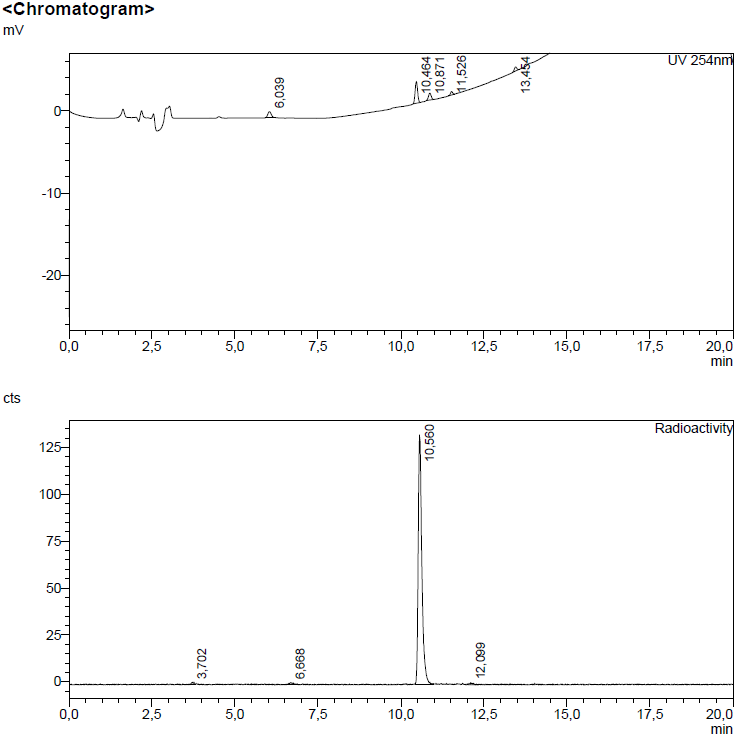


Figure S2. An example of a typical radio-HPLC chromatogram for [^11^C]EAI045
